# Economic evaluation of single-photon emission-computed tomography versus stress echocardiography in stable chest pain patients

**DOI:** 10.1038/s41598-022-19496-8

**Published:** 2022-09-08

**Authors:** Javad Javan-Noughabi, Aziz Rezapour, Marjan Hajahmadi, Vahid Alipour

**Affiliations:** 1 Social Determinants of Health Research Center, Mashhad University of Medical Sciences, Mashhad, Iran; 2 Department of Health Economics and Management Sciences, School of Health, Mashhad University of Medical Sciences, Mashhad, Iran; 3 Health Management and Economics Research Center, School of Health Management and Information Sciences, Iran University of Medical Sciences, Tehran, Iran; 4 Department of Cardiology, Rasoul Akram General Hospital, Iran University of Medical Sciences, Tehran, Iran

**Keywords:** Health care economics, Cardiology

## Abstract

The timely diagnosis of coronary artery disease (CAD) is an important medical problem. This study aims to assess the cost-effectiveness of Single-Photon Emission-Computed Tomography (SPECT) compared with stress echocardiography in stable chest pain patients. An economic evaluation study was conducted to assess the cost-effectiveness of SPECT versus stress echocardiography in stable chest pain patients without known CAD between April 1, 2017, and September 1, 2018 in Tehran, Iran. This study was performed from a societal perspective. The incremental cost-effectiveness ratio was calculated using a decision tree model. In addition, the robustness of results was examined by deterministic and probabilistic sensitivity analysis. This study showed that the expected cost and expected QALY for Stress echocardiography was $1106.75 and 0.83 respectively. Also, SPECT had expected cost and expected QALY equal to $1622.39 and 0.80 respectively. Finally, Stress echocardiography was the dominant strategy for CAD, with a lower cost and greater effectiveness than SPECT. The stress echocardiography can saved $18,528.17 per QALY. A deterministic and probabilistic sensitivity analysis confirmed the robustness of the results. Stress echocardiography was a more cost-effective method for diagnosing CAD disease in stable chest pain patients without known CAD compared to SPECT.

## Introduction

Nowadays, coronary artery disease (CAD) is recognized as one of the prominent threats to the health of human societies. In 2013, the disease was identified as the leading cause of death in the world, which was responsible for the death of more than 17.3 million patients. It is predicted that by considering the rapid growth of the disease in 2030, the mortality rate will exceed 23.6 million people^1^. In Iran, the annual incidence of coronary artery disease in men and women is estimated at 1436 and 1168 cases per 100,000 people, respectively^2^. In addition, coronary artery disease is responsible for about 156 deaths per 100,000 people in Iran^3^. Coronary artery disease (CAD) is not limited to mortality and disability, but also has important health and economic consequences^4^. By increasing the demand for medical care, an increase in health costs will be evident^4^. On the other hand, this issue may lead to reductions in productivity in the workplace due to absenteeism or poor performance of employees, which ultimately lead to low economic growth and intensify poverty and inequality^4^. According to studies, the cost of coronary artery treatment in the European Union was about 20 billion euros in 2009, and the reduction in production due to mortality and related disability is estimated at 18 billion euros^5^. In 2010, the direct costs of treatment and the loss of productivity due to cardiovascular disease are estimated at $109 billion in the United States, and by 2030 the cost of coronary artery disease is estimated to rise to $218 billion^5^. Therefore, coronary artery disease is predicted to be a major factor in the disability of economically active people, which can severely affect the productivity of active labor in society and reduce GDP and national income^4,5^. In addition, although the mortality rate of this disease has lately decreased with the advancement of science, the number of coronary artery patients is increasing who need to embrace a new specific lifestyle^6^. The consequences of this disease are early retirement, frustration, fear, anxiety, depression, lifestyle changes, decrease in life expectancy, and quality of life^7^.

According to what has been said, coronary artery disease is increasing in Iran and has many detrimental effects on the economic status and quality of life of patients. However, this disease is one of the most preventable chronic diseases by timely diagnosis. Invasive cardiovascular tests and diagnostic procedures account for a significant portion of the annual health care expenditures. Economists estimate that not investing in the prevention and treatment of coronary artery disease could cost $37,000 billion over the next 25 years^8^.

Therefore, cardiologists will use non-invasive methods and approaches in the first stage of the cardiovascular disease diagnosis, and in the following stages, they will use invasive methods such as angiography^9^. Non-invasive methods include electrocardiography (ECG), echocardiography (ECHO), exercise electrocardiography (Ex-ECG), cardiac single-photon emission computed tomography (SPECT), stress cardiac magnetic resonance imaging (C-MRI), exercise echocardiography (EX-ECHO), and stress echocardiography (stress ECHO)^10^. Among non-invasive methods, the use of a stress echocardiogram test is widespread in people who are unable to exercise, such as patients with peripheral vascular disease, heart attack, and orthopedic disorders^11^. The stress echocardiogram is not only a very powerful tool with extensive application, but also is an inexpensive technique for non-invasive cardiac imaging. This test can be performed quickly and versatility and ready availability, are its other advantages. In fact, it is a non-invasive tool for detecting myocardial ischemia by creating and recording stress-induced wall motion disorders. The accuracy of echo stress for detecting significant coronary stenosis is 80 to 90%, which is higher than exercise testing^12^. Stress echocardiography works similar to a CT scan, but the advantage of echo stress is a reduction in the patient radiation exposure over a CT scan^13^. According to studies, the SPECT (Single Photon Emission Computed Tomography) has also a high diagnostic value in coronary artery disease and has been proposed as a principal measure for patients^14^. However, there are significant differences between the two diagnostic methods in both cost and effectiveness. In a study conducted by Lee et al., it is showed that the medical direct cost for SPECT is $332. In another study, the cost of SPECT was $300 whereas the cost of stress echocardiography was $120. Although SPECT is much more expensive than stress echocardiography, it has shown better negative predictive value than stress echocardiography^15,16^.

In terms of cardiovascular patients’ follow-up, differences are reported between cost, sensitivity, and specificity of the SPECT method and the stress echocardiogram. In addition, various effects of treatment methods have been revealed on patients’ quality of life and preventing possible complications. However, due to the lack of conclusive clinical evidence in support of the dominant diagnostic method in all health systems, this study aimed to conduct an economic evaluation for comparing the SPECT method with the stress echocardiography in stable chest pain patients.

## Methods

We assessed the cost effectiveness of two different diagnostic strategies, SPECT and stress echocardiography, for stable chest pain patients without known CAD. This study was a quantitative economic evaluation that was performed at the three main tertiary hospitals in Tehran, the capital of Iran, in 2018. The present study was a census and no sampling was performed. This study included only those patients who had undergone angiography after SPECT or stress Echocardiography. We used a decision tree model to find the cost effective modality. The response of each diagnostic modalities (SPECT and stress Echocardiography) was positive or negative. The prognostic value of this modalities were assessed by invasive coronary angiography as a gold standard for all patients^17^. In this step, we had true positive, true negative, false positive and false negative response for each modality in decision tree model. There was 3 different treatments including CABG, PCI and drug treatment for patients with true positive and false negative results. There was no further process for patients with true negative and false positive.

The required data for the study included cost and utility data. In this study, cost data of diagnostic methods were collected from the perspective of the community. To collect cost data from the community perspective, direct medical and nonmedical costs and indirect costs were collected. Direct medical costs included: cost of diagnostic tests, angiography, physician's visit, nursing and counseling services, operating room, medicine, consumables, radiography, ECG, laboratory, hoteling, and other medical procedures. Direct nonmedical costs included: costs of travel, food, accommodation and telephone. It should also be noted that indirect costs were estimated using the human capital approach. Regarding how to calculate indirect costs, it's noteworthy that the lost productivity of the individual or his companions during the illness became the basis for calculating indirect costs. For this purpose, the number of days of absence from work of the person or his companions due to illness was multiplied by the amount of lost income of this period, and through this, indirect costs were calculated. Patients' records were used to collect direct medical costs, but direct nonmedical costs and indirect costs were collected through patients' interviews. All costs were converted to US dollars using the average exchange rate Central Bank of Iran for the time period of this study. According to this, one dollar is equal to 36,692 Iranian Rials.

In the current study, utility values were extracted using the European quality of life five-dimension scale. One of the reasons for choosing this questionnaire is its simplicity and tangibility for individuals, especially patients. In addition, the basis for calculating the desirability score is the preferences of the people in the community, and therefore it reflects the preferences and desirability of the health of the individuals in the community in a better way. Therefore, it is a suitable questionnaire for cost-effectiveness studies. The weights of the questions in this questionnaire have been estimated and localized by Goodarzi et al. for Iran^18^. According to the clinical specialist, the patients' desirability was measured one week after the necessary treatments through interviews with patients. Face to face interviews were conducted for completing the questionnaire and written informed consent was obtained from participants before data collection. This questionnaire includes five areas of mobility, self-care, routine activities, feeling pain or discomfort, anxiety or depression. Each of the questions (from 1 to 5) has three scales. A health rating scale was also used, which is rated from zero to 100, and individuals determine their current health status on it. According to previous studies, the validity, and reliability of this questionnaire for different components have been reported from 0.77 up to 0.88. The Cohen's kappa coefficient of this questionnaire for different components has been reported between 0.61 up to 1 in retesting^18,19^. Data related to the EQ-5D questionnaire were extracted from interviews with patients.

**Figure 1:**
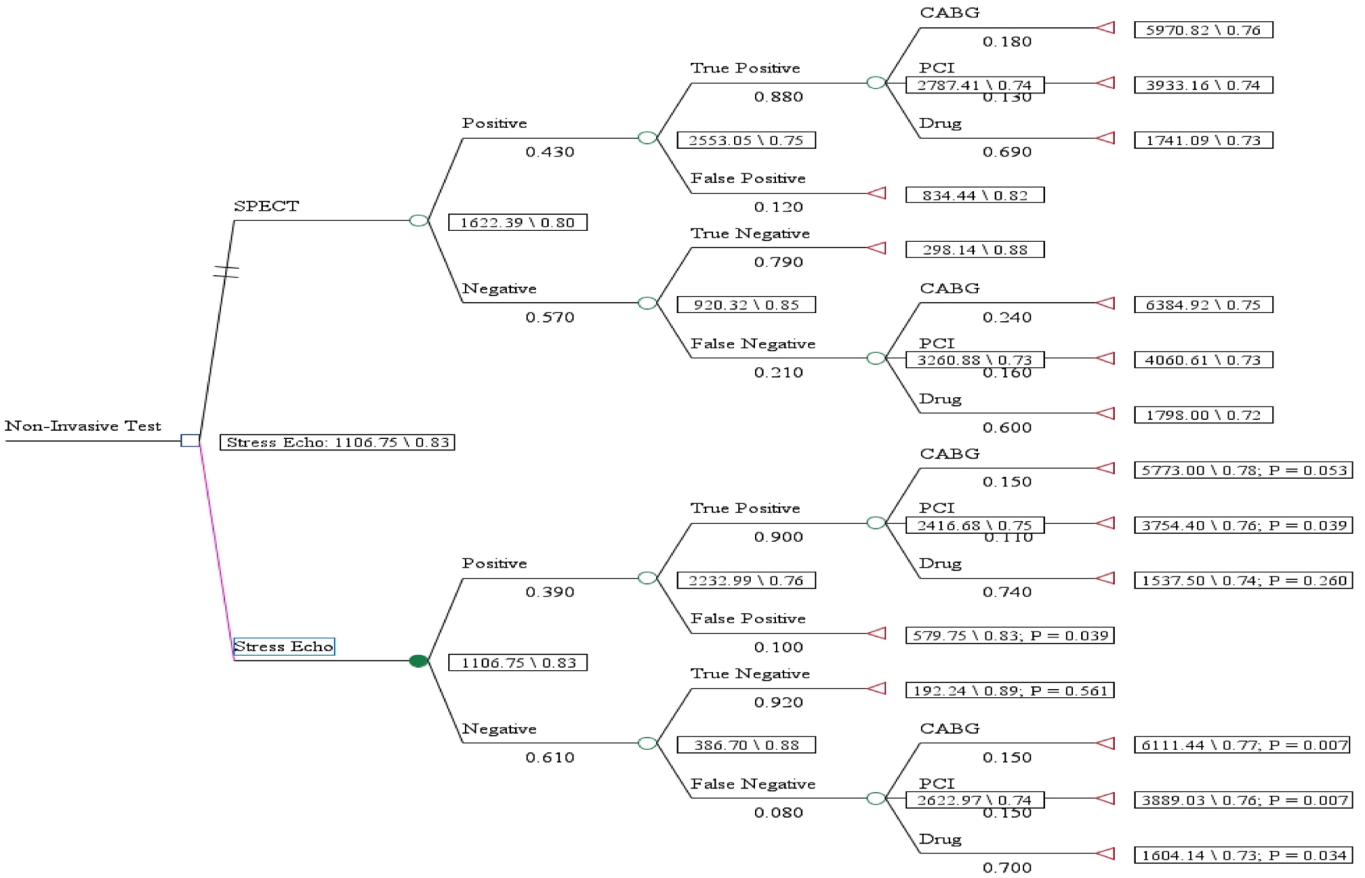
The results of the decision tree model.

Ultimately, data were analyzed by using Treeage software to calculate the cost-effectiveness of the SPECT diagnostic method in comparison to the stress echocardiography. The decision tree model of the cost-utility of the SPECT diagnostic method in comparison to the stress echocardiography is shown in the Fig. 1. All methods were performed in accordance with the relevant guidelines and regulations.

### Ethics approval

Ethical approval for this study was obtained from Ethics Committee of the Iran University of Medical Sciences.

### Consent to participate

Written informed consent was obtained from participants before data collection. All methods were performed in accordance with the relevant guidelines and regulations.

## Results

In this economic evaluation study, 207 patients used the SPECT diagnostic method and 303 patients used the stress echocardiography diagnostic method. The mean (± standard deviation) age of patients was 56.89 ± 53.16 years in SPECT group and 60.84 ± 56.11 in Stress echocardiography group. The majority of participants were women, married, and with non-university education. Table 1 shows the demographic characteristics of patients.

**Table1:** Demographic characteristics of patients.

Variation		Diagnostic modalities	
		SPECT	Stress echocardiography
Age		56.89 ± 53.16	60.84 ± 56.11
Sex	Men	76 (37%)	133 (44%)
	Women	131 (63%)	170 (56%)
Marital status	Married	114 (55%)	185 (61%)
	Single	93 (45%)	118 (39%)
Education	University education	89 (43%)	121 (40%)
	Non-university education	118 (57%)	182 (60%)
Current smoker		24 (12%)	46 (15%)
Diabetes		16 (8%)	31 (10%)
Hypertension		38 (18%)	55 (18%)
Total		207 (100%)	303 (100%)

The mean cost of CAD diagnosis and treatment in two groups of diagnostic modalities is compared in Table 2. This table shows that medical direct costs and non-medical direct costs in both groups were the highest and lowest cost respectively. As seen in Table 2, the surgical cost with US$ 323.33 was the highest type of medical direct cost in SPECT group and diagnostic cost with US$ 134.53 was the highest type of medical direct cost in Stress Echocardiography arm. Both non-medical direct cost and indirect cost were higher in the SPECT group than the Stress Echocardiography group.

**Table 2:** The cost items for CAD diagnosis and treatment.

Costs		Diagnostic modalities (Average costs)	
		SPECT	Stress echocardiography
Medical direct costs	Diagnostic	243.06	134.53
	Visits	49.46	17
	Surgical costs	323.33	101.10
	Consumables	224.78	69.17
	Drugs	104.84	38.49
	Laboratory	84.54	27.85
	Hoteling	221.11	69.98
	Total	1251.16	458.14
Non-medical direct costs		85.30	39.61
Indirect costs		283.68	114.60
Total		1515.31	612.35

Figure 1 shows the results of the decision tree model. In the structure of the decision tree model, the numbers below each branch indicate the transition probabilities. The percentage of patients in each branch was determined by following the patients of each group. The percent of true positive and true negative responses were 88% and 79% for SPECT group and 90% and 92% for stress echocardiography group. Payoffs including cost and QALY were calculated for patients in each branch. Then, payoffs were added at each of the terminal nodes of the decision tree. The run of the model shows that stress echocardiography was a cost-effective diagnostic option for CAD patients with a cost of $1106 and QALY of 0.83.

The results of cost-effectiveness rankings of the options have been indicated in Table  3. The results show that stress echocardiography was more effective and less costly versus SPECT. The incremental cost effectiveness (ICER) was $-18,528.17 per QALY.

**Table 3:** Cost effectiveness ranking.

Ranking	Strategy	Cost	Incremental Cost	Effectiveness (QALY)	Incremental Effectiveness	ICER	Result
1	Stress echo	1106.75	0	0.83	0		
2	SPECT	1622.39	515.64	0.80	− 0.0278	− 18,528.17	Dominated

**Figure 2:**
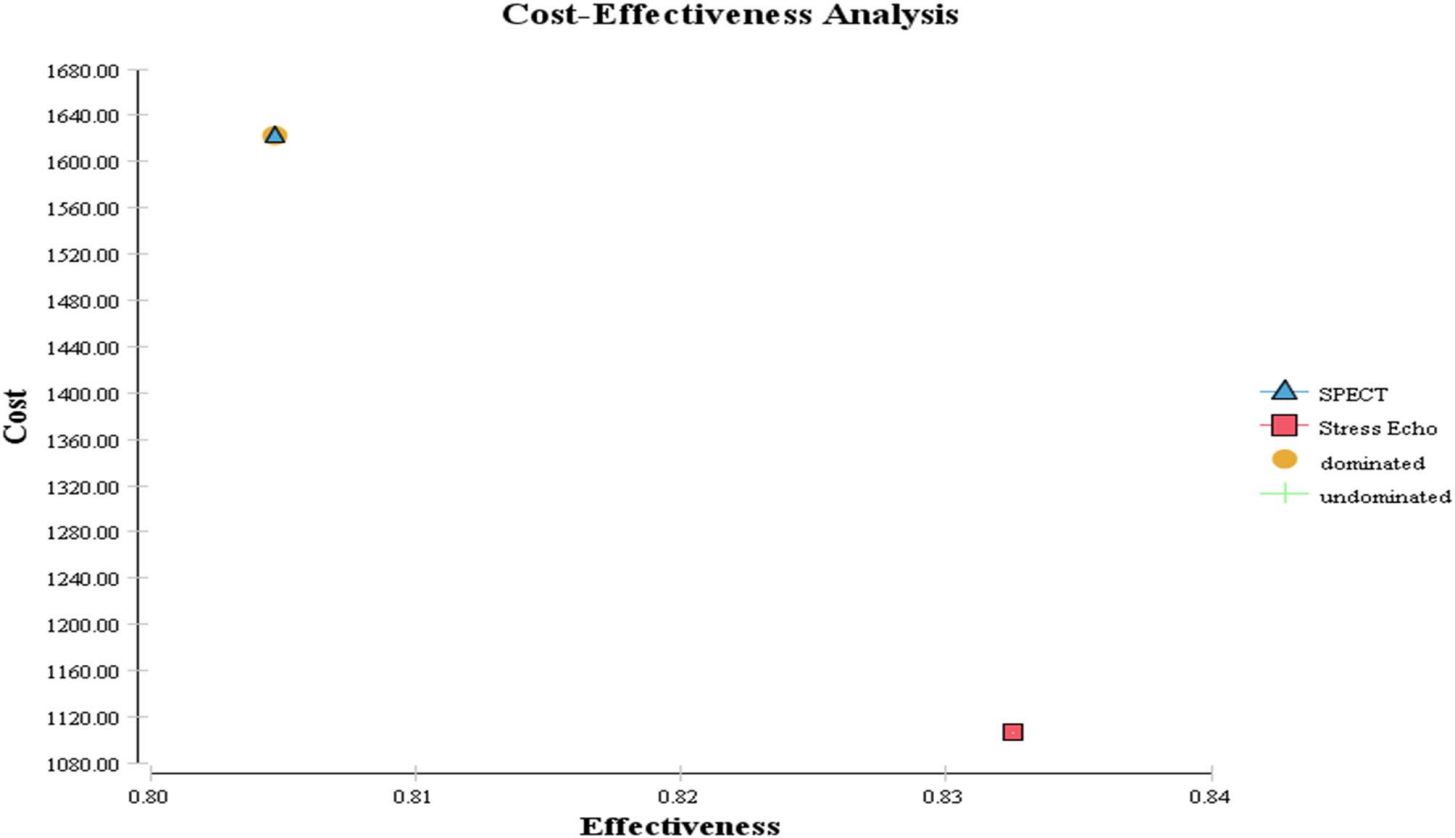
Cost effectiveness analysis of SPECT versus stress echocardiography.

The cost-effectiveness results of two diagnostic modalities were compared in Fig. 2. The dominant strategy, Stress echocardiography, is shown with a square in Fig. 2 (less costly and more effective). Also, SPECT as a dominated strategy is shown with the triangle (more costly and less effective).

### Sensitivity analysis

Deterministic and probabilistic sensitivity analysis were conducted to assess the robustness of the model to uncertainty in the parameters.

**Figure 3:**
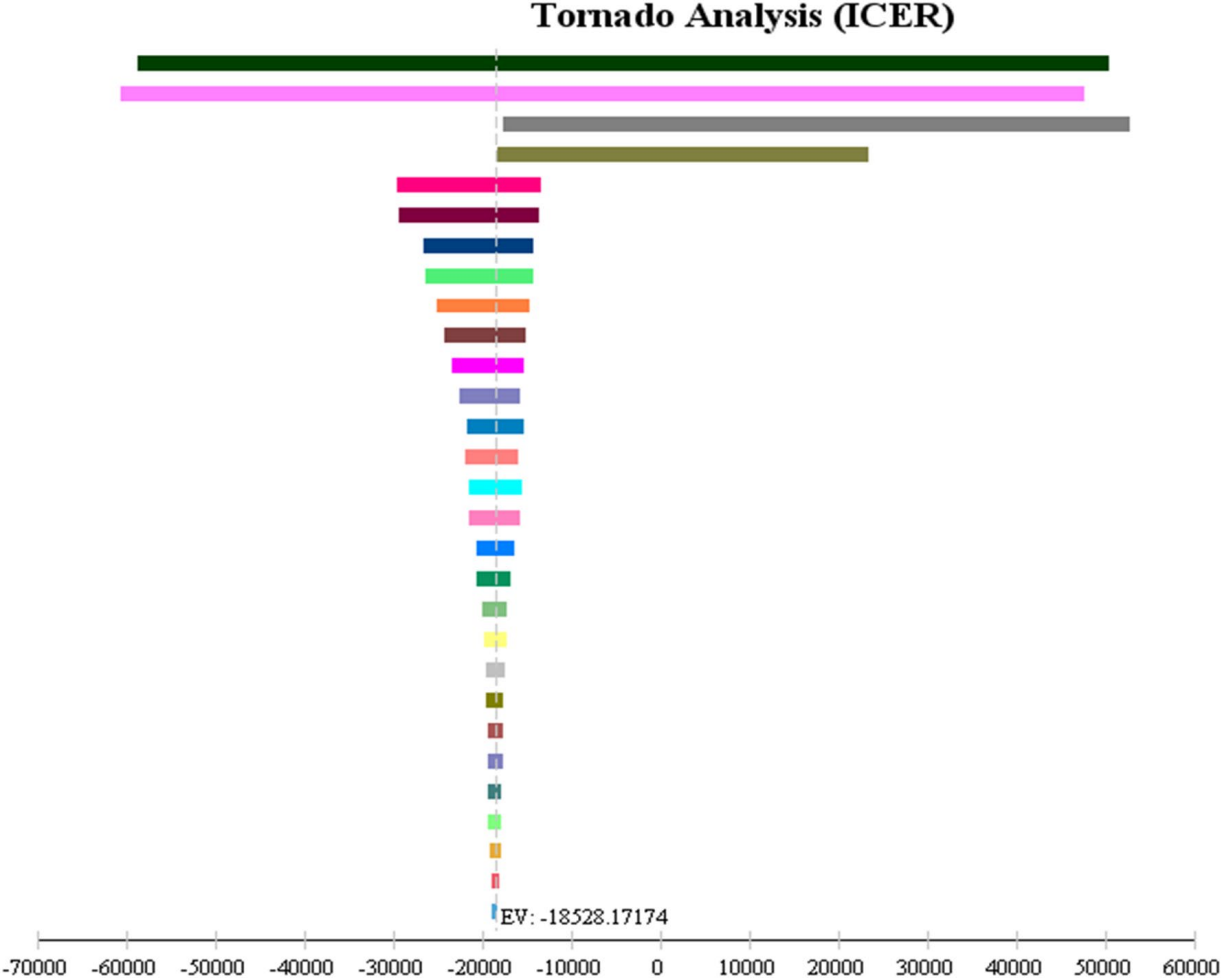
Tornado diagram for multiple one-way sensitivity analysis.

Multiple one-way sensitivity analyses were performed to assess the potential influences of different parameters to the incremental cost-effectiveness ratio (ICER), and the results are shown as the Tornado diagram. The Fig. 3 (Tornado diagram) showed that ICER has the highest sensitivity to changing the QALY of patients in the drug state with true positive response of the stress echocardiography test.

**Figure 4:**
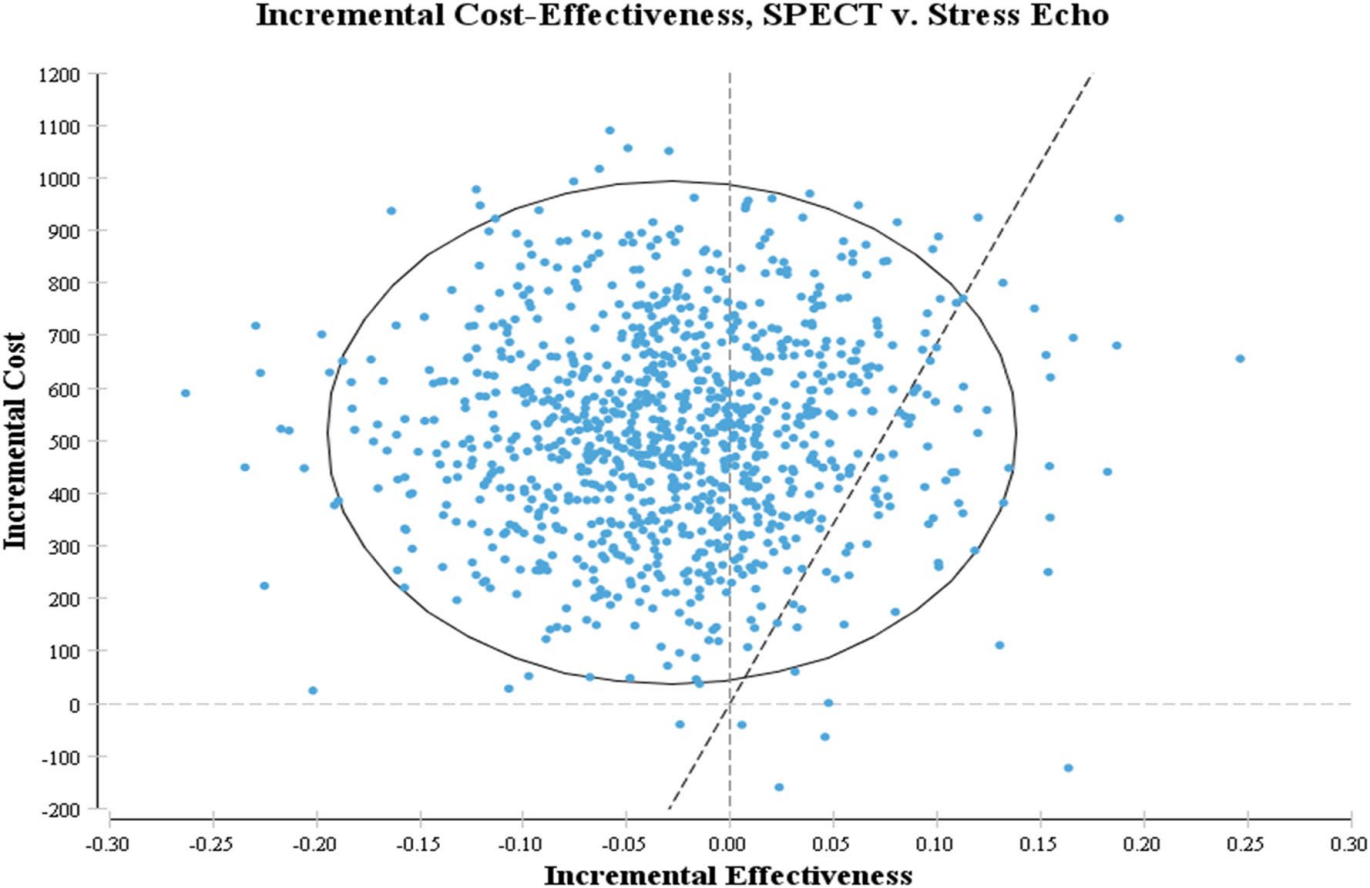
Incremental cost-effectiveness plane.

**Figure 5:**
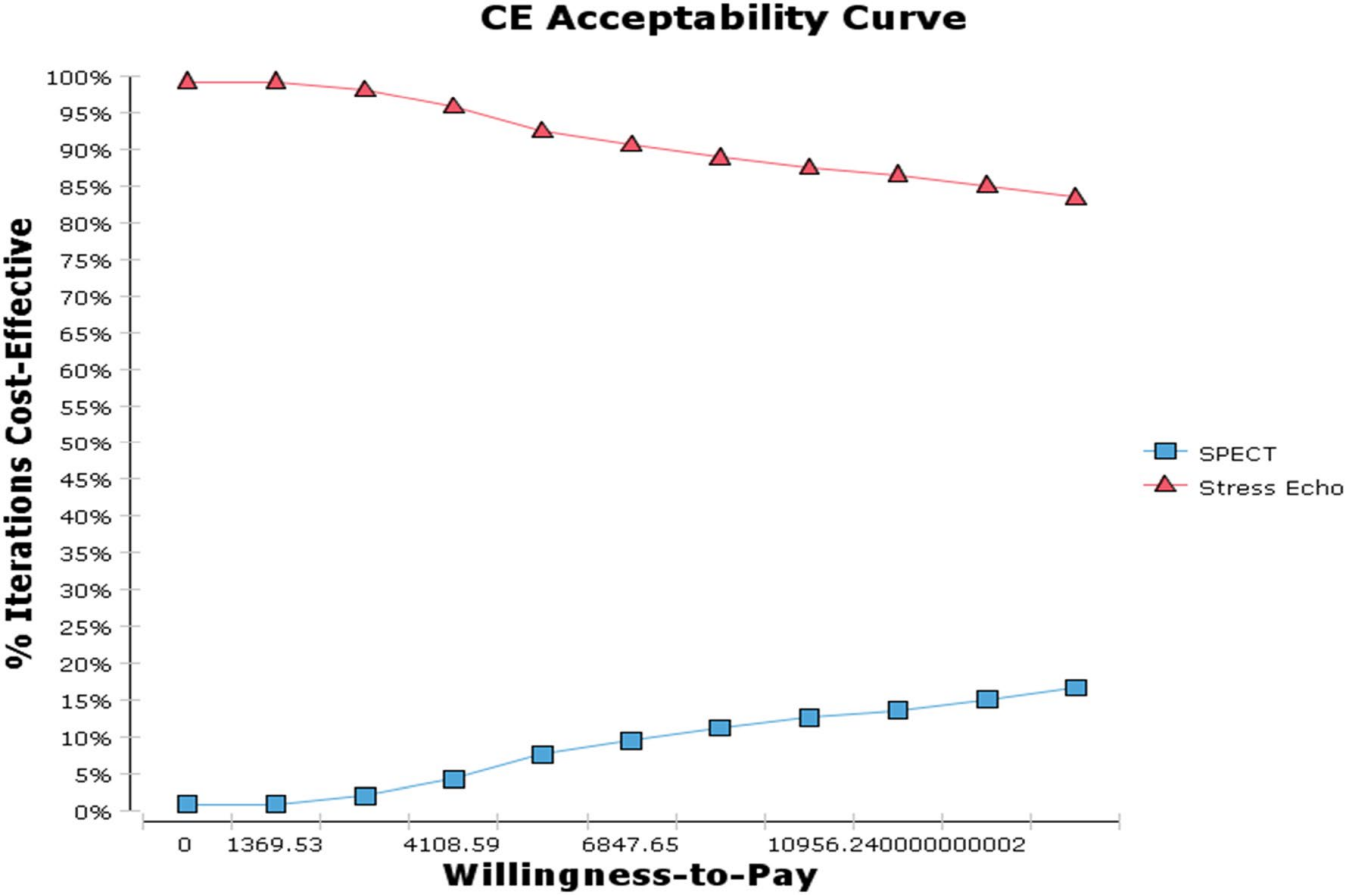
Cost-effectiveness acceptability curve.

Probabilistic sensitivity analysis are showed in the Figs. 4 and 5. As seen in Fig. 4, the results of Monte Carlo simulation of 1000 individuals confirmed the base case findings. Acceptability curves based on the results of Monte Carlo simulation (1000 patients) showed that stress echocardiography would achieve cost effectiveness for 92% of patients at a level of willingness-to-pay equal to $US6847.65/QALY.

## Discussion

To the best of our knowledge, our research is the first economic evaluation study to evaluate the cost-effectiveness of SPECT versus stress echocardiography from societal perspective in Iran.

Cost analysis showed that the cost of the SPECT diagnostic test was US$ 243.03, meanwhile the cost of using the stress echocardiography method for CAD diagnosis was US$ 134.53. Also, total medical direct costs per patient (the costs of diagnosis plus treatment) were $1251.16 in the branch of SPECT and $458.14 in the branch of stress echocardiography. Both of the non-medical direct costs and indirect costs in the branch of SPECT was more than stress echocardiography. Lee et al. in a study entitled “Cost-effectiveness of coronary CT angiography in patients with chest pain: Comparison with myocardial single photon emission tomography” showed that the medical direct cost for SPECT is $332. In this study, the cost of angiography was estimated about $601^16^. In another study, the cost of SPECT was $300 whereas the cost of stress echocardiography was $120, and for coronary angiography was $1200^15^. Hlatky et al. in an economic outcomes study calculated the costs of Coronary Anatomy Imaging modalities. In this study, the costs of SPECT, PET and computed tomography angiography (CTA) were calculated for 2-years. The most expensive and cheapest diagnostic modalities were PET and SPECT respectively. The mean costs of PET, CTA and SPECT for 2-years were $6,647, $4,909 and $3,965^20^. Van der Wall et al. conducted a cost analysis study on diagnostic modalities (including CTA, PET and SPECT) for diagnosis of suspected CAD. This study showed that SPECT is much more cheaper than CTA and PET over 2 years follow-up of suspected CAD patients^21^. However, it is reported that SPECT is significantly more expensive compared with stress echocardiography for CAD diagnosis^22^.

The result of this study showed that stress echocardiography (with more effectiveness and lower cost) is more cost effective than SPECT in diagnosis of CAD in stable chest pain patients without known CAD.

The deterministic sensitivity analyses for the ICER confirmed the robustness of the base case. Moreover, a probabilistic analysis demonstrated efficiency at a WTP threshold of $6847.65 per QALY gained in 92% of the iterations.

Based on the findings of cost-effectiveness analysis, the diagnostic method of stress echocardiography was dominant over the SPECT. In this study, the SPECT method reduces the utility by 0.028 and increases costs by $515.64 in comparison with the Stress echocardiography and as a result is known as a dominated strategy. A systematic review revealed that SPECT is an attractive strategy compared with PET and CTA. But, SPECT in comparison with stress echocardiography, DECT and CMR was a dominated modality^23^. A study by Ferreira et al.^22^ in Portugal, revealed that the cost of stress echocardiography ranged from $36 to $64 and the cost of performing the SPECT diagnostic procedure ranged from $60 to $92^22^. In their study, the SPECT and stress echocardiography have been compared as the diagnostic tests of CAD. In this study, in which the cost-effectiveness criterion was “cost per diagnosis accuracy,” the sensitivity and specificity of the two tests were investigated. Finally, they concluded that both SPECT and Stress echocardiography tests were dominated by angiography^22^.

In another study, the cost per QALY for the SPECT method was estimated at $38,000 to $40,316, which was much higher than the stress echocardiography method^24^. The findings of this study are consistent with the findings of the present study. It is showed that combined non-invasive strategies with computed tomography coronary angiography (CTCA) and stress imaging are cost effective for the diagnosis of stable coronary artery disease^25^. The study by Lee et al., conducted in South Korea, also examined the cost-effectiveness of the SPECT diagnostic test in comparison with the angiography. In this study, two criteria of “cost per diagnosis accuracy” and “cost per QALY” were used to perform economic evaluation. When “cost per diagnosis accuracy” was used as cost-effectiveness criteria, angiography was introduced as the dominant cost-effective strategy over the SPECT method. However, when the “cost per QALY” was used as criteria of cost-effectiveness, it has been concluded that for people with a pretest probability less than 60%, the SPECT method was more cost-effective^15^.

One of the limitation of this study was that we included only those stable chest pain patients without known CAD who had undergone angiography after SPECT or stress Echocardiography.

## Conclusions

According to the results, stress echocardiography was more cost effective than SPECT to diagnose CAD. The current study has significant concepts for decision-making in designing clinical guidelines for the diagnosis of CAD in stable chest pain patients without known CAD.

## Data Availability

The datasets used and/or analyzed during the current study are available from the corresponding author on reasonable request.
